# Projecting the Long-Term Impact of School- or Community-Based Mass-Treatment Interventions for Control of *Schistosoma* Infection

**DOI:** 10.1371/journal.pntd.0001903

**Published:** 2012-11-15

**Authors:** Xiaoxia Wang, David Gurarie, Peter L. Mungai, Eric M. Muchiri, Uriel Kitron, Charles H. King

**Affiliations:** 1 Department of Mathematics, Case Western Reserve University, Cleveland, Ohio, United States of America; 2 Center for Global Health and Diseases, Case Western Reserve University, Cleveland, Ohio, United States of America; 3 Division of Vector Borne and Neglected Tropical Diseases, Ministry of Public Health and Sanitation, Nairobi, Kenya; 4 Department of Environmental Sciences, Emory University, Atlanta, Georgia, United States of America; 5 Schistosomiasis Consortium for Research and Evaluation, University of Georgia, Athens, Georgia, United States of America; College of Public Health and Health Professions, United States of America

## Abstract

**Background:**

Schistosomiasis remains a significant health burden in many areas of the world. *Morbidity control*, focused on limiting infection intensity through periodic delivery of anti-schistosomal medicines, is the thrust of current World Health Organization guidelines (2006) for reduction of *Schistosoma*-related disease. A new appreciation of the lifetime impact of repeated *Schistosoma* infection has directed attention toward strategies for greater suppression of parasite infection *per se*, with the goal of *transmission interruption*. Variations in drug schedules involving increased population coverage and/or treatment frequency are now undergoing field trials. However, their relative effectiveness in long-term infection suppression is presently unknown.

**Methodology/Principal Findings:**

Our study used available field data to calibrate advanced network models of village-level *Schistosoma* transmission to project outcomes of six different community- or school age-based programs, as compared to the impact of current 2006 W.H.O. recommended control strategies. We then scored the number of years each of 10 typical villages would remain below 10% infection prevalence (a practicable level associated with minimal prevalence of disease). All strategies that included four annual treatments effectively reduced community prevalence to less than 10%, while programs having yearly gaps (‘holidays’) failed to reach this objective in half of the communities. Effective post-program suppression of infection prevalence persisted in half of the 10 villages for 7–10 years, whereas in five high-risk villages, program effects on prevalence lasted zero to four years only.

**Conclusions/Significance:**

At typical levels of treatment adherence (60 to 70%), current WHO recommendations will likely not achieve effective suppression of *Schistosoma* prevalence unless implemented for ≥6 years. Following more aggressive 4 year annual intervention, some communities may be able to continue without further intervention for 8–10 years, while in higher-risk communities, annual treatment may prove necessary until eco-social factors fostering transmission are removed. Effective ongoing surveillance and locally targeted annual intervention must then become their mainstays of control.

## Author Summary

Debate persists about how best to prevent disease caused by *Schistosoma* parasites. Current guidelines focus on suppressing morbidity by limiting average intensity of infection during childhood. However, this may not be sufficient to cure infection or prevent reinfection, leaving risk for sub-clinical morbidities such as growth stunting and anemia. More intensive programs involving broader coverage and/or more frequent treatments could potentially cure most infections and even prevent their return. Because such programs' effectiveness is not currently known, we used computer simulation (grounded by past treatment program results) to project short- and long-term impact in communities where *Schistosoma* are common. We estimated that 4 annual treatments (delivered community-wide or only to school-age children and high-risk adults) could effectively reduce local prevalence below 10%. Programs with gap years were less effective, particularly in high-risk communities. If a program was successful, infection stayed <10% for 7–10 years in low risk communities. However, rapid resurgence (within 1–5 years) was projected for high risk villages. We conclude that, given the networked transmission of *Schistosoma*, annual treatment programs of sufficient duration can have secondary benefits, *i.e.*, long-term suppression in some areas. However, high risk areas will need continuing surveillance and frequent retreatment to truly minimize their risk for disease.

## Introduction

Schistosomiasis is an environmentally transmitted parasitic disease that results in increased morbidity and mortality among millions of people living in tropical and subtropical regions [1]–[3]. Different control initiatives have had success in reducing either the prevalence or the mean intensity of *Schistosoma* infections in a number of individual countries [4]–[9] although, for other settings, the potential effectiveness of such strategies remains mostly unknown. With combined approaches of snail control, chemotherapy, health education, and hygienic improvement, China has made substantial progress against *S. japonicum*, which has been successfully eliminated in several provinces [10]–[13]. In Brazil, mass chemotherapy programs have achieved at least a 50% to 70% decrease in *S. mansoni* prevalence, with a marked reduction in infection-related morbidity and hospitalization in many states [14]. However, the effects of those initiatives have been uneven because of the regional- and country-specific differences in local transmission, in available resources, and in overall program commitment [12], [15].

In the meantime, schistosomiasis remains a major public health problem in sub-Saharan Africa [3], [8]. Possible measures for controlling African schistosomiasis include chemotherapy (primarily praziquantel therapy), snail control (by mollusciciding or habitat modification), provision of safe water alternatives, and/or education with behavior modification to limit exposure [4]. Among these, chemotherapy-based mass treatment remains the most widely used at present [8]. ‘Morbidity control’, focused on limiting infection intensity through periodic delivery of anti-schistosomal medicines, is the thrust of the World Health Organization (WHO) schistosomiasis control guidelines [16]. However, a new appreciation of the lifetime impact of repeated *Schistosoma* infections [17], [18] has directed attention toward strategies for greater suppression of parasite infection *per se*, with the ultimate goal of transmission interruption as a more effective elimination of disease risk. However, experience in schistosomiasis control programs suggests that current broad-based drug delivery campaigns may not consistently reduce local prevalence or prevent rapid reinfection [19], [20].

Randomized field trials are now underway to examine the relative impact of increased population coverage and/or treatment frequency in community-wide or school age-targeted treatment programs for *Schistosoma* control [21]. However, their effectiveness relative to existing WHO protocols is not yet known. In the present study, we used a computer simulation based on a recently developed, *stratified worm burden (SWB) model* [22] of multi-village *Schistosoma* transmission to project programmatic success for each of seven different mass treatment control strategies in a typical *Schistosoma*-endemic region of sub-Saharan Africa.

## Methods

### Predicting the burden of *Schistosoma* infection based on a Stratified Worm Burden model

In Gurarie, et al. 2010 [22], we developed a new approach to predicting schistosomiasis transmission and community-level infectious burden using models based on a *Stratified Worm Burden* (SWB) formulation, combined with calibration using data from a completed multi-year pilot schistosomiasis control program. In this approach, a host population is subdivided into burden strata in terms of individual parasite load, with the *i^th^* stratum carrying (*i*−1) Δ*w* to *iΔw* worms, where the size of the stratum, *iΔw*, is prescribed *e.g.*, at increments of an additional 10 worm pairs per person. The transitions among strata depend on (i) force of infection (determined by the transmission environment and human risk factors); (ii) worm attrition (natural or drug-induced); and (iii) human demographics (birth, aging, mortality, growth sources). Details are presented in Supplement S1 and [22].

### Advantages of SWB modeling

The strength of this SWB model analysis is that it more accurately reflects many of the underlying complexities of *Schistosoma* species transmission, while yielding a more usable projection of community infection *prevalence* (rather than mean worm burden) after a campaign. The SWB includes and tracks differences in infection risk between children and adults, the highly skewed distribution of infection intensity among infected individuals, and the networked meta-population aspects of transmission among neighboring villages sharing common water sources [23]. This is an advantage where decision-making policy is made on the basis of local population or school age prevalence of *Schistosoma* infection [16] After the model was calibrated with available field data from pilot control programs [24], [25], it was used to project the likely outcomes of different targeted- or population-wide mass treatment programs now being introduced into endemic areas in sub-Saharan Africa [21], [26]. The model can realistically account for inevitable program limitations in program resources, delivery efficiency, etc., whereby drug treatment cannot cover every infected human in the treatment area and the frequency of the treatment cannot be too high. Non-stationary and nonlinear models of this type are difficult or impossible to solve through classical equation analysis. However, their performance and outputs can be estimated for different chemotherapy scenarios through numerical analysis [22] in the programmed simulations presented here.

**Figure 1:**
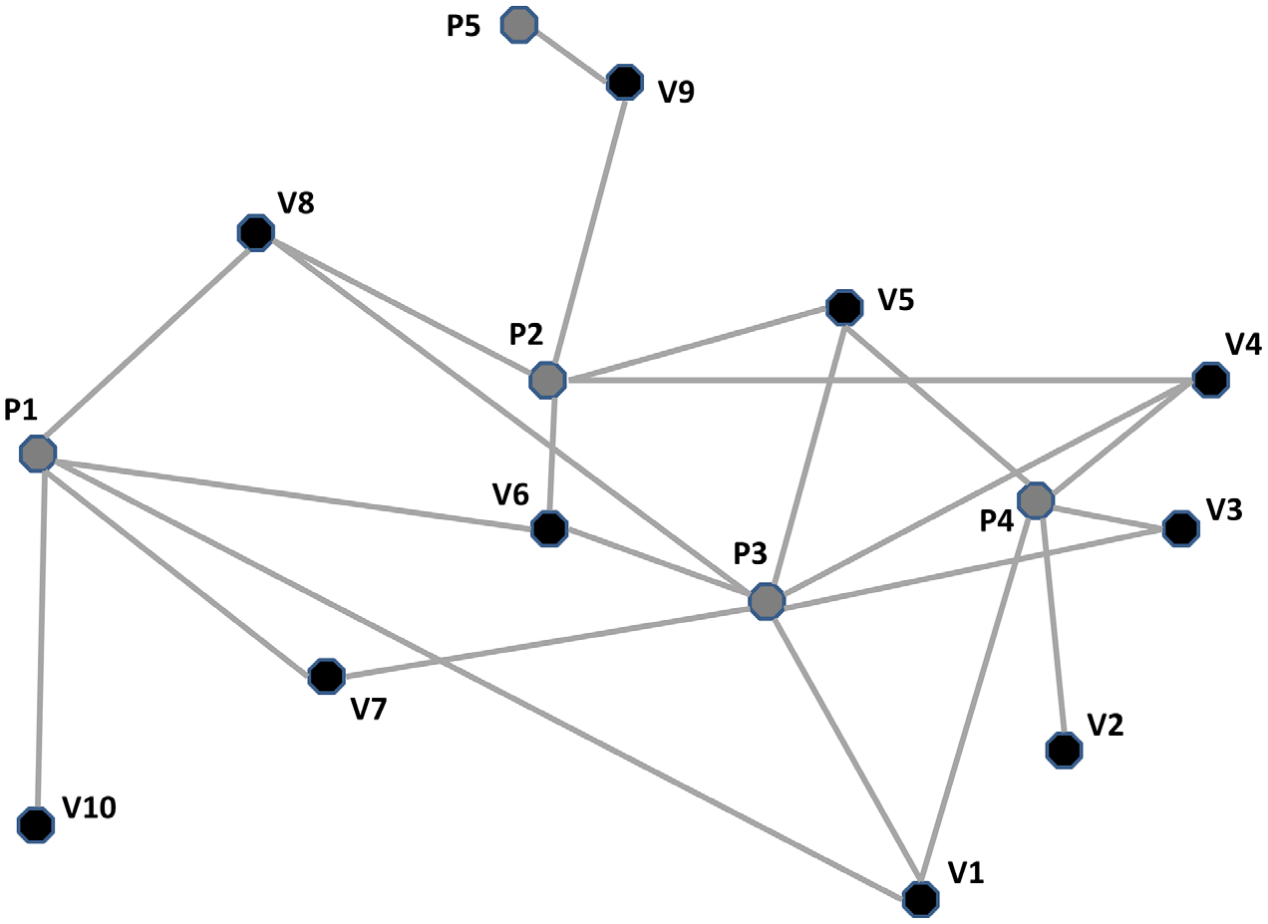
Schematic view of a networked landscape of *Schistosoma* transmission. The diagram indicates the typical overlapping transmission links (gray lines) between human habitation sites (villages V1–V10, black dots) and their respective primary and secondary water contact sites (ponds P1–P5, gray dots) where people become exposed to cercariae from infected host snails. While each village may have its own principal transmission characteristics, this linked meta-population structure can foster more rapid reintroduction of infection in previously treated communities [22], [23].

Taking burden strata as model variables instead of the population mean worm burden, SWB carries detailed information of the infection status of a host population and reflects its highly aggregated distribution (typical of *Schistosoma* and other helminth infections [27]), without imposing *a priori* assumptions about that distribution. Statistical moments (variance and/or aggregation) of such a distribution can include human population dynamics and, in its most simplified format, even reflect the predictions of the classical Mean Worm Burden (MWB) equations of MacDonald [28]. Following the SWB approach, we were able to develop calibration procedures [22] that could readily take advantage of available prevalence and intensity data from published treatment trials [24], [25]. The calibration methodology was, first, to estimate local force of infection in each village through fitting of SWB equilibrium equation parameters to prevalence data, then using reduced MWB and snail prevalence systems to estimate transmission parameters and the net effects of other unmeasured biotic and abiotic variables associated with transmission at local water sites [22]. These local factors include, in aggregate, water quality, rainfall, vegetation, seasonal water level persistence, human contact and sewage contamination. This model development allowed prediction of treatment outcomes across distributed village systems (Figure 1), including the significant effects of having multiple human age-strata sharing multiple water contact sites.

For the present study, our base-case study system consisted of 10 neighboring villages and 5 shared water (snail) sites, where the village level infection exposures are linked through the shared water sites (see Figure 1 for schematic representation of the transmission network). The calibration is taken from a well-studied *Schistosoma haematobium*-endemic region of the Msambweni District in coastal Kenya [24], [25], [29]–[31]. The 10 villages used to calibrate this study are located 50 km southwest of Mombasa, Kenya in an agricultural region that produces rice, coconuts, sugar cane, cassava, and maize. The area has a monsoon-type climate, with the period of January to April–May being hot and dry. The mean monthly temperature varies from 26.3°C to 26.6°C, with lows of 23.5°C (July) to 26.9°C (March–April) and highs from 26.7°C (August) to 36.3°C (February). Annual precipitation varies from 900 mm to 1,500 mm, with large yearly and monthly fluctuations. There are usually two rainy seasons, the long rains starting around March and continuing until July, and the short rains beginning in October and lasting through November. In 1984, the total population of this area was determined by household census to be 8,957. It was 16,790 by repeat census in 2002–3. Surveys of water use in the villages indicates that exposure to cercariae-infected waters comes through the practice of using dammed streams and seasonal ponds for washing and bathing, despite the availability of piped water (from kiosks) and borehole wells in most villages. No other schistosomiasis control programs were implemented in the study area during the course of these studies; routine chemotherapy dispensed by the local hospital was monitored, and there was no interval change in the level of such treatment.

As is typical for schistosomiasis [32], pre-treatment levels of *Schistosoma* infection school age prevalence varied significantly (23% to 74%) across the landscape, even within a span of 5 km [33]. Data available for model calibration included: (i) demographic data, *i.e.*, human population numbers in different villages divided into child and adult age groups and, in addition, snail population densities at georeferenced exposure sites [34], [35]; (ii) infection, in terms of individual and mean egg counts for children in each village, and also the density of susceptible, infected, and shedding adult snails in all monitored water sites [34], [35], [36]; (iii) behavioral data on water contact exposures for each village population among their adjacent water contact/snail-infected sites [24], [31]. Full human and snail data were collected in 1983–1987 and again in 2000–2003 [24], [31], [33]–[35]. Human prevalence data for 2 villages were collected in 2006 and 2009 [37]. In all human surveys infection prevalence and mean egg-counts were based on standard 10 mL filtration of two urine samples [38] collected from the resident populations surveyed. The transmission coefficients from snail sites to villages and from villages to snail sites and the infection (egg excretion) rates for children and adults were estimated based on model fitting to these data. The calibrated parameters in [22] consisted of *per capita transmission rates* A (snail-to-human) and B (human-to-snail), partitioned among different human villages *i* = {1,2,…,10}, snail sites *j* = {1,…,4}, and demographic groups (“children”, adults”). All other inputs (demographic, environmental) are then entered into the model as additional factors. For instance, having human population (“children”+“adult”) *H_i_* = *H_i_^c^*+*H_i_^a^* at village “*i*”, that carry MWB {*w_i_^c^, w_i_^a^*}, and snail number/infection prevalence {*N_j_, y_j_*} at site “*j*”, we estimate the forces of infection between “i” and “j” as
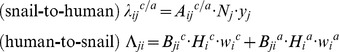
in terms of per-capita rates {*A_ij_^c/a^*}, {*B_ji_^c/a^*}. The overall force of human infection at each site *λ =  λ^c/a^*, along with “young/adult” demographics, will determine the SWB distribution for each group through equilibrium relations (see Fig. 2 of [22]).

Our basic assumption is that per-capita rates remain unchanged in time. So having calibrated model with the 1983–1987, and 2000–2006 data, we can apply it then for any other period, changing demographics and starting infection levels *ad hoc* for a given time period. Because local environment remains stable, where transmission decreases it is due to the reduced number of infected humans. For the current analysis, we have taken 2009 data for demographics and human infection [37] as the starting point (initial model inputs) and have numerically simulated the effect of different treatment strategies over long (multi-year) periods, taking into account projected population changes (growth parameters {*H_i_^c/a^, N_j_*} - as prescribed functions of time), grounded on most recent human census and snail recovery trends.

### SWB estimation of mass-drug administration effects

The effect of drug treatment on the prevalence of *Schistosoma* infection was implemented in the model by instantaneously shifting humans at higher burden levels (stratum) to lower levels according to the established efficacy of praziquantel [25], [39]. As an example, within the model, a treatment session with an efficacy of 90% (*i.e.*, killing 90% of worms) would bring a person in the 10^th^ stratum, who is carrying between 90 and 100 worm pairs before therapy, down to the 0^th^ strata (carrying 0 to 10 worms), as the remaining number of worm pairs is expected to be between 9 and 10 of worms after the treatment. Because egg output at this level of infection is, in practice, undetectable [40], all persons in the 0^th^ stratum are considered ‘uninfected’, at least in terms of their contribution to transmission risk. In each year, community percent prevalence is estimated as [1−(fraction in the 0^th^ stratum)]×100. This is a more realistic effect than modeled in the past, in which a treatment term has typically been added to the natural mortality of worms to indicate an extra loss. In fact, the drug-killing effect on worms occurs more quickly (in less than one month) than natural death (∼4–5 years [27]), and this more flexible scheme allows better prediction for the various options in timing and coverage among suggested control strategies [16], [21]. In our model's simulations we assumed at-random treatment coverage, in the sense that a random subset of each population was treated each time [41], [42]. In this sense, the ‘coverage’ of a control program means the fraction of all people treated in each treatment session, regardless of their past participation. Annual population growth for study villages was estimated to be 1.5%, based on the smoothed averages for household census surveys taken in 1984, 1987, 2000, 2003, and 2006.

### Strategies compared for mass-drug administration of anti-schistosomal chemotherapy

Currently, the ‘standard-of-care’ for population-based drug treatment of *Schistosoma haematobium* and *S. mansoni* is based on the latest 2006 WHO guidelines [16]. Under these guidelines, populations are divided into 3 classes according to local prevalence of infection among children. These are: high-risk communities (>50% children infected), moderate-risk communities (10–49% infected), and low-risk communities (<10% prevalence), respectively (Table 1). For highly infected population, it is recommended that children between 5 and 15 year old, as well as adults, be treated annually; for moderately infected populations, children between 5 and 15 yr of age and high-risk adults are treated on an every 2 year schedule; for populations with low prevalence of infections, it is recommended that school children be treated twice, upon their entry into school and at school completion (Table 2).

**Table 1:** 2006 World Health Organization categorization of communities at risk for schistosomiasis [16].

Community category	Prevalence among school-aged children:
I. High-risk community	≥50% by parasitological methods (intestinal and urogenital schistosomiasis), or ≥30% by questionnaire for visible hematuria (urogenital schistosomiasis)
II. Moderate-risk community	≥10% but <50% by parasitological methods (intestinal and urogenital schistosomiasis), or <30% by questionnaire for visible hematuria (urogenital schistosomiasis)
III. Low-risk community	<10% by parasitological methods (intestinal and urogenital schistosomiasis)

**Table 2:** WHO 2006 recommendations for population-based treatment of at-risk communities according to prevalence category [16].

Community category	Intervention	Health services and community-based intervention
I. High-risk Community [prevalence ≥50%]	Treat all school-age children (enrolled and non-enrolled) once a year	Also treat adults considered to be at risk (from special groups to entire communities living in endemic areas)
II. Moderate-risk community [prevalence 10 to 49%]	Treat all school-age children (enrolled and non-enrolled) once every 2 years	Also treat adults considered to be at risk (special risk groups only)
III. Low-risk community [prevalence <10%]	Targeted treatment of school-age children (enrolled and non-enrolled) twice during primary schooling age (e.g., once on entry and once on exit)	Praziquantel should be available in dispensaries and clinics for treatment of suspected cases

### Ongoing operational research on schistosomiasis control: the Schistosomiasis Consortium for Operational Research and Evaluation (SCORE) treatment trials

Since 2010, new large-scale operational research trials have been initiated in seven schistosomiasis-endemic locations in sub-Saharan Africa, situated in Cote d'Ivoire, Niger (2 sites), Kenya (2 sites), Tanzania, and Mozambique [21]. These studies seek to identify more cost-effective approaches to regional and national schistosomiasis control, within the present-day framework of mass treatment campaigns for helminth control [26], [43]. The randomized SCORE trials involve implementation in 25 villages per study arm, and will compare the relative costs and effectiveness of different drug delivery strategies for population-based control of either *S. haematobium* or *S. mansoni* infection. Two different kinds of SCORE studies will compare relative effectiveness of program implementation in communities having either low-moderate or higher prevalence of infection, defined as 10–24% prevalence among school-age children (low) vs. ≥25% (high), respectively. For the selected SCORE villages, there was village-level randomized assignment to one of several different treatment coverage and delivery options: Some communities will receive community-wide treatment (CWT), which will include treatment for all consenting adults as well as school age children; other communities will receive the more standard school-age targeted treatments (SBT) along the lines of current W.H.O. recommendations [16]. With each type of delivery, some communities will receive yearly therapy, some will transition from CWT to SBT mid-way through the project, while others will transition to every other year treatments or to ‘drug holidays’. CWT will involve the most extensive treatment coverage, aiming to include as many people as possible, whereas SBT will target schools (with non-attenders of school age also encouraged to participate), while drug holiday means there will be no treatment for the year. For communities with high infection prevalence (>25%), there are 6 proposed strategies over the 4-year study period:

1. CWT–CWT-CWT-CWT [C-C-C-C]
2. CWT-CWT-School based-School based [C-C-S-S]
3. CWT-CWT-Holiday-Holiday [C-C-H-H]
4. School based-School based-School based-School based [S-S-S-S]
5. School based-School based–Holiday-Holiday [S-S-H-H]
6. School based–Holiday-School based-Holiday [S-H-S-H]

For populations with lower infection prevalence (10 to 24%), no CWT is included in the SCORE trials, and treatment will be either school–based every year [S-S-S-S], two years of SBT followed by two year holiday [S-S-H-H], or SBT every two years [S-H-S-H], with resulting community prevalence determined in the 5^th^ year.

An important consideration in choosing to implement a more intensive drug treatment programs will be the incremental cost-effectiveness relative to standard-of-care [44]. For this paper's analysis, we focused on two major costs: the cost for community screening (in determining the prevalence of active *Schistosoma* infection in the population) and the cost of drug treatment (drug costs+delivery costs). In the SCORE program, screening and assignment is performed only at the beginning of the 4 year treatment regimen, so that screening costs are the same in each arm. However, for comparison among current WHO-recommended strategies, we have also modeled the possible impact of rescreening and reassigning treatment regimens on a yearly basis, and for the current WHO approaches (not actually researched by SCORE) we estimated the potential cost-savings and change in effectiveness using repeated rapid screening among school age children and reassignment of treatment strategies based on those annual results [45]–[47]. [Although it requires extra expense, screening before treatment has the potential to reduce the drug cost by adapting to a less expensive strategy when prevalence of infection becomes less intense.] We took the cost of initial community sensitization and screening to be approximately one U.S. dollar (USD) per person, based on in-country costs documented by the Partnership for Child Development and other national control programs [48], The cost of annual drug dose was estimated at 0.25 USD per child and 0.50 USD per adult. Rapid program screening is typically carried out by sampling 50–100 persons for each village [45], so 100 USD was the maximum estimated screening cost per each village, no matter how large the population. The calculated cost for drug depended on the number of people treated, so information on total population, coverage for each treatment strategy, and number of villages treated was tailored to the intended coverage of each specific strategy. Taking the end of 2009 as the model's time baseline, we then projected the following metrics of each drug treatment strategy: i) the number of years needed to bring down the every village prevalence to a low-risk level (<10%) and ii) the number of years a village was likely to remain at this safe level following the termination of treatment.

## Results

### The problem of reinfection—‘reworming’ after deworming

**Figure 2:**
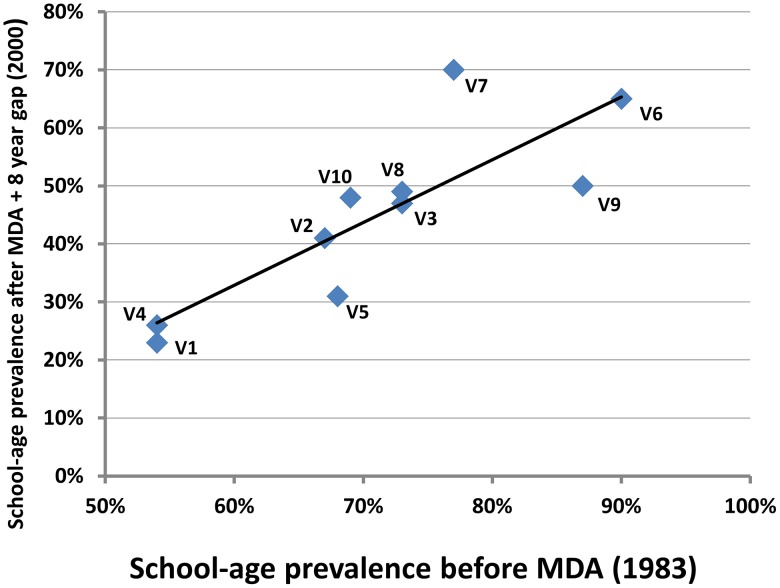
High correlation between pre- and post-treatment prevalence of *Schistosoma* infection at the local village-level. Village-level *S. haematobium* prevalence values for school-age children are plotted for 10 neighboring communities included in the Msambweni Study in coastal Kenya [24], [25], [29]. Pretreatment prevalence values from Fall 1983 are indicated on the x-axis, while post-treatment values for 2000 are indicated on the y-axis. During the 1983–2000 interval, the participating communities received school-based targeted drug administration from 1984–1992, after which a funding lapse led to suspension of treatment. The 2000 values thus indicate a robust return of infection prevalence after an 8 year hiatus of control. Pearson correlation (R = 0.83, *P*<0.001) indicated a strong association between pre- and post-control village-level prevalence values. Corresponding linear regression (y =  1.08x−0.32) indicated that the post control prevalence was, on average, 32 percentage points lower than before control. Nevertheless, all villages relapsed to the moderate or high *S. haematobium* prevalence categories, with pre-control prevalence an excellent predictor of the level of post-treatment rebound. These and post-treatment data from additional yearly surveys were used to calibrate the SWB model used in this study.

One of the limitations of treatment delivered in population-based deworming campaigns is the risk of reinfection, sometimes referred to as ‘reworming’ [49]. The complex nature of *Schistosoma* parasite transmission can leverage parasite persistence in both snail and human hosts, while incomplete adherence with program-delivered treatments allows for persistent egg contamination by untreated residents [50]. Our previous analysis of a multi-year school-based drug treatment program for *S. haematobium* control indicated that the median time to reinfection can vary significantly depending on village of residence [20], [24]. Over an 8 year observation period (1983–1991), median time to reinfection could vary significantly between 2 to 8 years in adjoining villages [20]. The transmission potential within each community appeared fairly resilient to the ‘perturbation’ caused by targeted drug administration in schools, where ‘time since treatment’ and the specific study year did not significantly alter annual reinfection hazard [20]. Further, in 2000, we observed that after an 8-year period during which control had lapsed, communities that had had the highest levels of infection in 1983 (before any therapy had been given) were the same ones that had the highest levels of infection after the 8-year pause (Figure 2) (Rank test rho = 0.927, p<0.01). While, in general, school-age prevalence in each community, when we revisited in 2000, was about 32 percentage points lower than pre-control values, three of ten communities had reverted to the WHO ‘high prevalence’ category (≥50%, see Table 1 for definitions), and the remaining seven remained in the ‘moderate prevalence’ group (10–49%), while none were in the desired low prevalence category (<10%) associated with lowest morbidity risk after 8 years without control (Figure 2).

### Model calibration and validation for the present study

**Figure 3:**
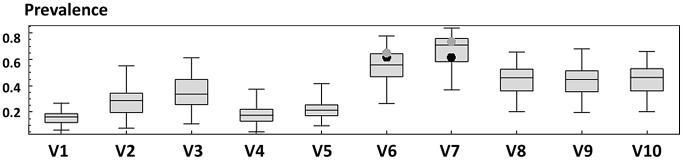
SWB model validation: prediction of 2009 village-level *S. haematobium* prevalence among school-age children. Following initial calibration of the programmed SWB model against human and snail *S. haematobium* prevalence data from 1983–1987 and 2000–2006 [22], we checked the predictive accuracy of the model against new survey data obtained for two study villages in 2009 after a round of community-wide treatment in 2006. The observed *S. haematobium* prevalence among school-age children (y-axis) is indicated by black dots for villages 6 and 7. The corresponding gray dots indicate the predictions of the model for these two villages using the model's best-fit parameter values. The box-plots indicate the median, interquartile range, and 95% range of the SWB model predictions in sensitivity analysis, in which model input parameters were allowed to vary at random over a ±20% range. The concordance between observed and predicted values in this and other validation testing [22] provided support for the accuracy of model projections in the present study simulations.

Prior to predictive simulation of treatment program outcomes, we used the most recent 2009 infection prevalence data from the same region of Kenya to check the accuracy of the SWB model's predictions about infection prevalence following perturbation by community-based drug therapy. Treatment interventions were previously implemented in that region in 2000, and on a limited basis in 2003 and 2006 [33], [51]: in 2000, 79% of those infected in the 10 village area were treated; in 2003 and 2006, only the most heavily infected villages, villages 6 and 7, were treated, with a coverage of 41–53% of all those infected in the area. Our new data from follow-up 2009 surveys [37] showed that the six-year post-treatment prevalence infection among children was again high: 61.7% for village 6 and 62% for village 7. Figure 3 shows the comparison of observed 2009 prevalence (black dots) and model predicted 2009 prevalence point estimates (gray dots) among school age children for villages 6 and 7. In sensitivity analysis, allowing model input parameters to vary randomly across a range of ±20% of their base-case values, observed 2009 prevalence values were well enclosed by the inter-quartile range of model outputs, indicating that its predictions were not extremely sensitive to changes in base case input parameters in this setting, and that our model had good predictive accuracy for this setting.

### Modeling area-wide implementation of WHO 2006 treatment guidelines

**Figure 4:**
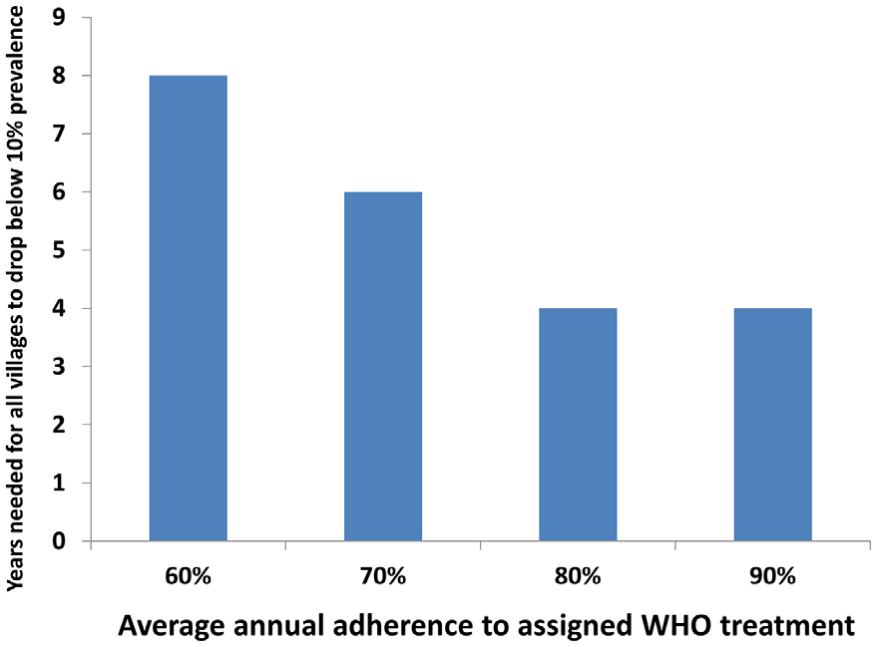
Modeling time-to-success for current WHO treatment strategies, as a function of program adherence. In this SWB model simulation for the 10 interlinked villages in Figure 1, WHO treatment assignment was made based on current recommendations for high- and moderate-prevalence communities (see Table 2). The bars indicate the number of years required for the overall area school-age population prevalence to drop below 10% (y-axis). This working target is a prevalence level is felt to be associated with minimal risk of advanced schistosomiasis [16]. As shown in the different bars along the x-axis, the time to needed to achieve the <10% global prevalence target varied according to the population participation (adherence) with the implemented treatment program. At 80–90% adherence among children (achieved in many research control programs), it took a minimum of 4 years of treatment (as assigned in year 1, based on starting prevalence) to achieve the <10% target. Where adherence was less good (60–70%, typical of many non-research national programs), control was projected to take appreciably longer (6–8 years on the assigned treatment strategy).

We next used our calibrated SWB model to project the likely outcomes of implementing the current WHO treatment guidelines (Tables 1 and 2) for high-, moderate-, and low-risk communities in the networked 10-village *Schistosoma*-endemic area (Figure 1). Using inputs based on recent community prevalence, we projected the number of years that would be required to bring all communities to a safer, low level prevalence category (<10%), as a function of community uptake (treatment adherence) across the program (Figure 4). In doing so, we had each community continue on its original treatment strategy assignment until *every* community achieved the <10% prevalence level. In our simulations, coverage for high-risk adults among high-prevalence populations was assumed to be at least 60%. As our model was able to target different burden strata, we interpreted “high risk groups” as those adults in the 5 highest worm burden strata. Figure 4 shows our model's estimates of the number of years the WHO regimen would have to be implemented to bring all villages under control as a function of adherence among treated children. At high levels of uptake (80–90% adherence) control was achieved in 4 years, whereas at lower levels of adherence (60%) control took twice as long (8 years).

We next asked whether repeated annual community screening and reassignment of treatment strategies (again based on the WHO recommendations in Table 2) could offer a more cost-effective approach to infection control. Table 3 indicates the results of following a rescreen/reassignment strategy for up to 8 years in the same communities. Whereas continuation of the originally assigned treatment regimen was capable of lowering prevalence to <10% among children in each individual community, the strategy of reassigning treatment regimens based on yearly community screening was much less effective. As noted in Table 3, the rescreen/reassign approach was unable to effectively suppress infection prevalence below 15%, even at the highest level of treatment uptake (90% adherence), likely related to the early switch to less intensive coverage as prevalence dropped. If adherence were less good (i.e., at a level of 60%), continuation of initial treatment strategies was less costly and more effective than the rescreen/reassign strategy. Notably, at that level of coverage, the final school age prevalence with the rescreening and reassignment approach was projected to remain at 35% at the end of 8 years (*i.e.*, well shy of the 10% goal). If, however, adherence were >70%, rescreen/reassign became a less costly option but with, again, conspicuously subpar effects on suppression of infection prevalence (Table 3).

**Table 3:** Performance of WHO 2006 strategies *with* or *without* yearly pretreatment screening with possible treatment reassignment.

		Rescreen and reassign village treatment regimen to a different frequency and/or coverage?					
		No	Yes	No	Yes	No	Yes
Coverage	Years of treatment	Total treatment units (villages treated per year_×_years of treatment)		Projected overall prevalence among children after the multi-year treatment program is completed		Total cost (in US Dollars) for drugs and delivery, ± additional screening	
0.6	8	48	32	9%	35%	$8173	$10028
0.7	6	36	24	7%	28%	$7490	$7470
0.8	4	24	17	8%	25%	$6295	$5164
0.9	4	24	17	4%	15%	$7081	$4725

### Effects of infection rebound after a multi-year, mass treatment intervention

**Figure 5:**
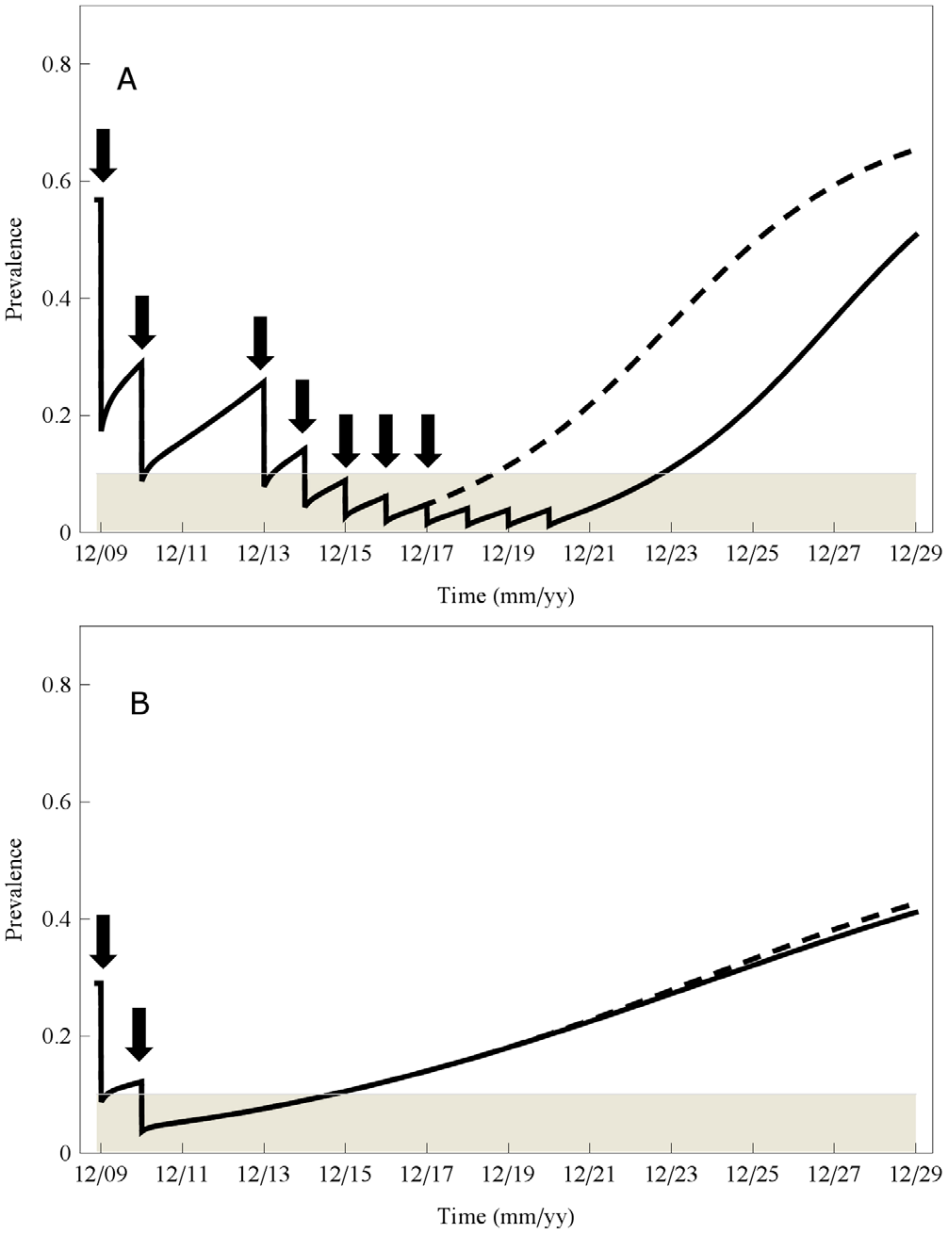
Projected rebound of infection in villages V2 and V6 after community-based treatment for two years. Graphs indicating the projected 20-year annual infection prevalence experience of two area villages, one of initially high prevalence (village 6, panel A) and one of moderate prevalence (village 2, panel B) during and after a 2-year annual community-wide drug administration campaign followed by a 2-year treatment hiatus (C-C-H-H). The timing of initial treatments is indicated by downward arrows. In the high-prevalence village (panel A), community coverage at 70% adherence among children and 50% adherence among adults reduces school age prevalence below the desired 10% target (gray zone) in two years. However, subsequent implementation of a treatment ‘holiday’ for 2 years results in rapid return of school age prevalence to moderate-level prevalence. At that point, re-implementation of treatment through an annual school age coverage results in continued suppression of prevalence to the <10% level. However, if school age treatment is again suspended after 4 years (dashed line) or 8 years (solid line) of retreatments, childhood prevalence is projected to slip above 10% within 3 years. By contrast, in panel B (lower prevalence Village 2), school age prevalence is adequately suppressed by 2 years of community-based treatment, after which local school age prevalence is expected to remain below 10% (gray zone) for at least 4 years. Here, dashed and solid lines indicate that the duration of school age treatment of village 6, whether for shorter (dashed line) or longer periods (solid line), has only marginal effect on the rebound of infection prevalence that is expected in Village 2.

In examining the success of WHO treatment regimens of differing frequencies and coverage, it was apparent that village level force of transmission (*i.e.*, its reinfection potential) played a large role in whether the program achieved or failed to achieve the goal of <10% prevalence. Local transmission factors also influenced whether a successfully treated community would stay in the ‘safe zone’ of <10% prevalence for any significant period of time after the mass treatment campaign was ended. Figure 5 demonstrates the projected prevalence outcomes for treatment of two modeled villages, one of high initial prevalence and continuing high transmission potential, and one of moderate prevalence and lower risk of reinfection. Both communities respond well to the first two rounds of drug therapy, quickly dropping to <10% prevalence. However, village A relapses to >25% prevalence after a 3 year hiatus in mass treatment, and requires ‘consolidation’ treatments to regain the <10% status. If consolidation is suspended after 4 years of annual treatments, infection prevalence rebounds to the moderate level (>10%) in two years. If consolidation runs for 8 years, community prevalence approaches it lowest levels, yet prevalence is expect to rebound over 10% in 3–4 years. By contrast, in the moderate prevalence village B having lower transmission potential, once local prevalence is well suppressed (after the second round of mass CWT treatment) then prevalence is expected to remain in the ‘safe’ zone (<10%) for 4–5 years. In this case, suppression of infection is used to indicate a persistent reduction of prevalence below 10% (the WHO-recommended threshold for low-risk communities), but this does not imply an interruption of transmission.

**Figure 6:**
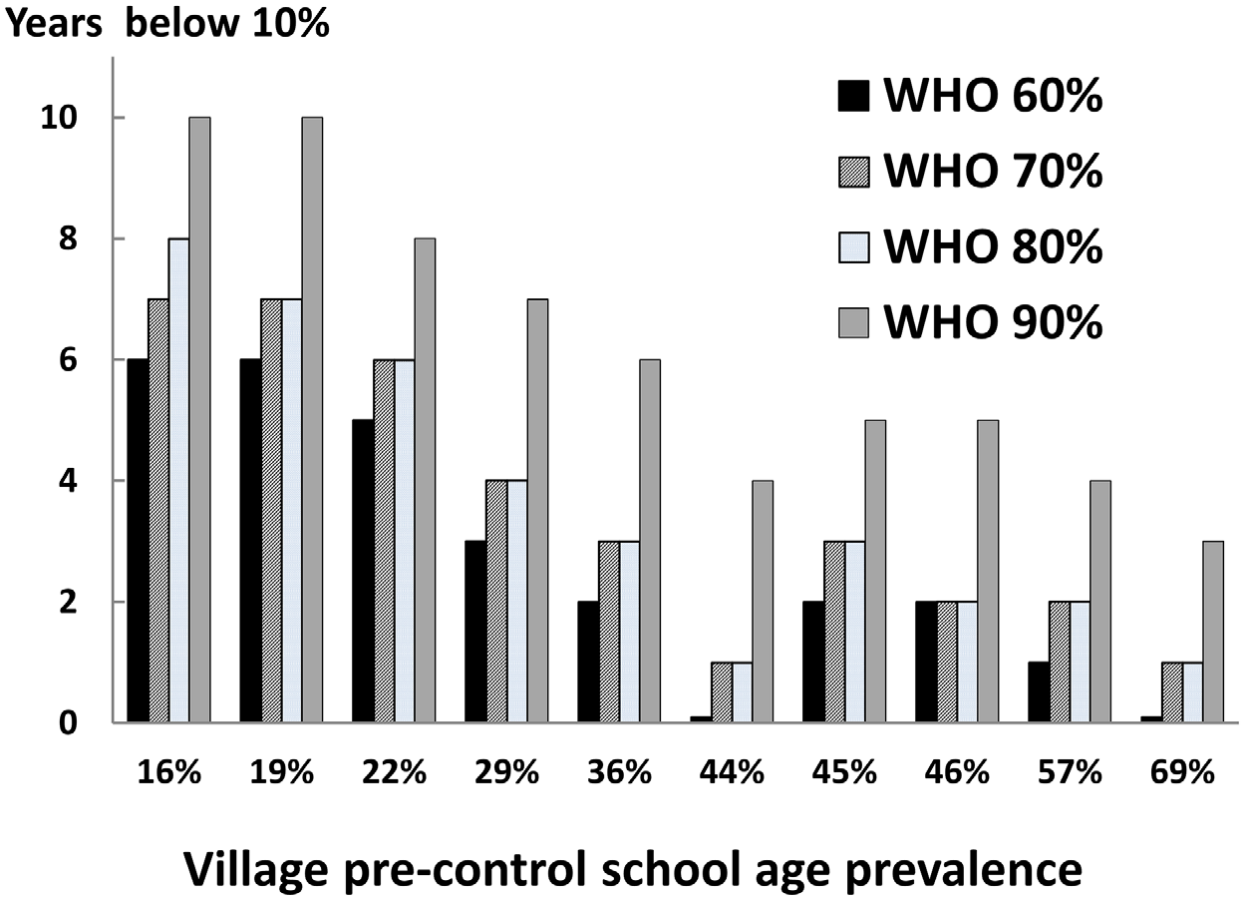
Likely impact of treatment adherence on long-term suppression of village prevalence using current WHO strategies. In this simulation, village-level outcomes are projected in terms of the number of post-treatment ‘safe’ years (i.e., years with school age prevalence <10%) that are likely if assigned treatment is ended once an areas achieves a <10% prevalence in all villages. Here, control is achieved using standard WHO treatment assignments, with the differently shaded bars indicating different levels of treatment adherence. (As detailed in Figure 4, such control requires 8 years of treatments when adherence is 60%, 6 years at 70% and 4 years at 80–90% adherence.) Of note, the duration of control varies strongly depending on each village's pre-intervention level of school age infection prevalence. Where adherence is low, the initially high-prevalence villages very quickly rebound to >10% prevalence and associated risk for disease. Where overall coverage is much higher (90%), every village is projected to have at least 3 years of ‘safety’ below 10% school age prevalence. Independent of adherence levels, the 5 villages with the lowest initial prevalence levels were projected to have at least 2 ‘safe’ years after the suspension of program-based treatment.

Figure 6 indicates the relative likelihood that a program based on WHO-recommended treatment schedules (without reassignment) would lead to successful long-term suppression of infection prevalence after mass treatment is suspended. The duration of continued suppression varied substantially between the modeled villages, and was much shorter in the communities with the highest levels of starting prevalence and greater links to high risk water sites (6–10 years suppression in lower prevalence communities *vs.* zero up to 3–4 years suppression in the highest risk communities). Results also varied the considerably according to the local adherence to treatment (60–90%, Figure 6). Where adherence was quite high (90%) suppression of infection with program intervention was much greater, suppressing local prevalences down to <5% (Table 3). Following this more aggressive level of suppression, rebound of infection prevalence (to >10%) took substantially longer in all villages (Figure 6).

### Projected outcomes for the SCORE program regimens

**Figure 7:**
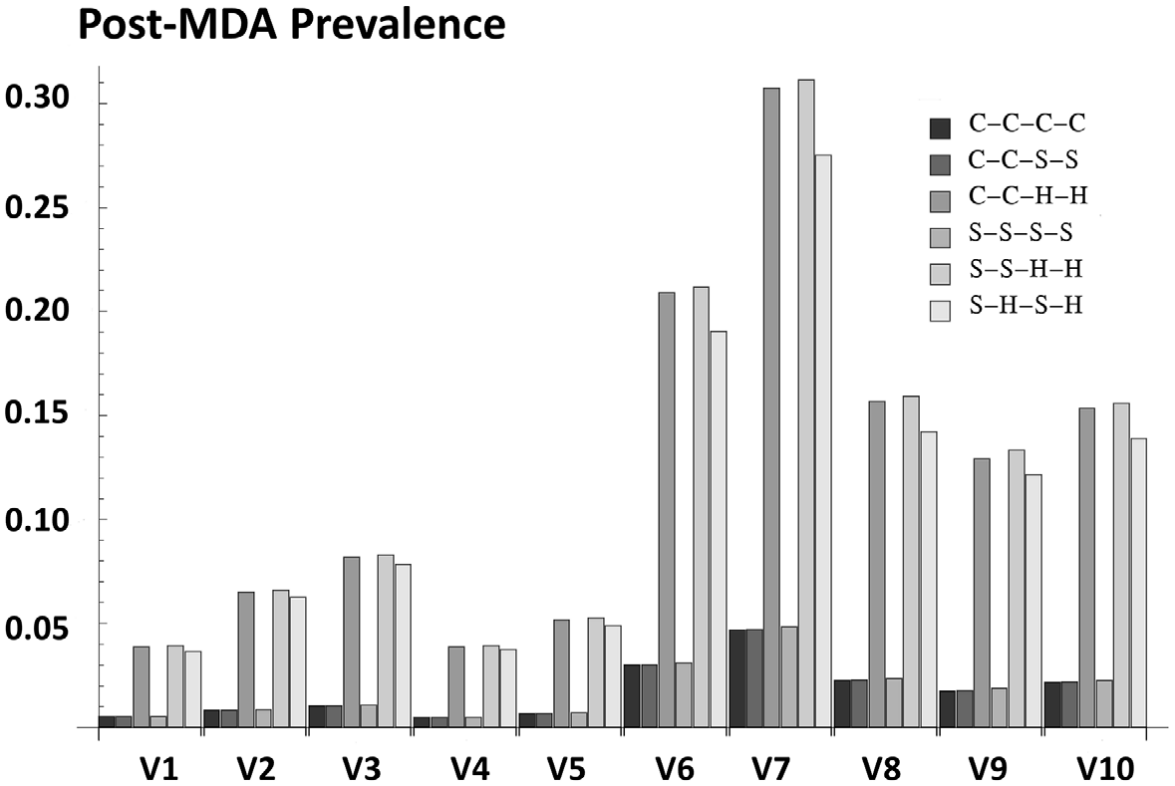
Projected impact of different SCORE treatment strategies on village level prevalence following 4 years intervention. Large randomized trials of different schedules of anti-schistosomal drug delivery are now underway, under the auspices of the Schistosomiasis Consortium for Operational Research and Evaluation (SCORE) [21]. In this simulation, we examined the likely outcomes of the six different SCORE control strategies on village level prevalence at after 4 years of treatment. School age adherence was set to 70%, and in community-based treatments, adult adherence was assumed to be 50%. C indicates a year of community-based treatment, S indicates a year of school age treatment, and H indicates a ‘holiday’ or non-treatment year. Of note, in the low-prevalence villages, all strategies achieved the goal of reducing village prevalence <10%. Control was obtained in the high prevalence villages only when there were no gaps in treatment, i.e., C-C-C-C, C-C-S-S, or S-S-S-S, with no ‘holidays’ in the schedule.

We next examined the potential of the six modified treatment regimens currently being researched by SCORE, in terms of their ability to achieve and maintain very low prevalence of *Schistosoma* infection. Final outcomes were quite different in the participating villages following 4 years of treatment in community-based or school-age targeted programs (including strategies having community = >school-age crossover in coverage (C-C-S-S), and those with gaps in treatments in different years (‘drug holidays’), i.e., C-C-H-H, S-S-H-H and S-H-S-H). Figure 7 shows the prevalence of each village after four years participation in each of the 6 candidate strategies. Strategies C-C-C-C, C-C-S-S, and S-S-S-S brought the prevalence of *all* villages well below the 10% prevalence objective after 4 years' treatment. By contrast, strategies C-C-H-H, S-S-H-H, and S-H-S-H achieved <10% in only half of the modeled villages, and these ‘successful’ villages were the villages with starting school-age prevalences of ≤36%. The projected prevalence outcomes at the end of C-C-C-C, C-C-S-S, or S-S-S-S treatment were similar, suggesting that addition of treatment for adult populations had relatively limited incremental benefits in terms of reducing local prevalence of infection. Strategies C-C-H-H, S-S-H-H, and S-H-S-H were not able to bring all villages to a safe level of infection, with post-program prevalences of 12–31% in 5 out of 10 targeted villages after the 4-year intervention period, so further treatment would be necessary in these villages (as in discussed earlier for Figure 5).

**Figure 8:**
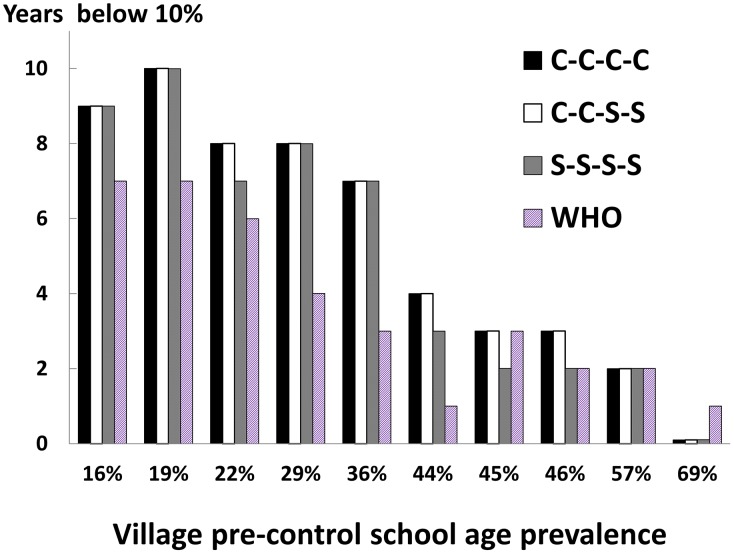
Likely impact of different SCORE strategies on long-term suppression of village prevalence of *Schistosoma* infection. As in Figure 6, we simulated the duration of long term post-control suppression of *Schistosoma* infection to safer levels below 10%. In this case, overall school age adherence was estimated at 70%, and comparisons were made between the 3 most successful four-year SCORE strategies, and standard implementation of the WHO strategy (which takes 6 years to achieve area control). The more aggressive, every-year SCORE strategies resulted in more prolonged post-treatment suppression of infection prevalence than the WHO protocols (in which some communities get assigned to every-other-year treatment). This was apparent in the six villages with the lowest pre-treatment prevalences (16–44%). In the villages with highest pre-intervention levels of infection (57–69%), all of the strategies were very limited in their ability to effect post-treatment suppression (0 to 2 years only).

Figure 8 indicates the projected number of ‘safe years’ (local prevalence <10%) after the 4 yearly rounds of successful area-wide C-C-C-C, C-C-S-S, or S-S-S-S intervention at an adherence level of 70%. At this level of adherence, each of these three strategies resulted in more post-program low prevalence years than projected for the six-year WHO 2006 strategy (for the latter, when assigned according to initial high/moderate/low prevalence category and *without* rescreening/reassignment in subsequent years). This difference was noted in the six villages having the lowest pre-treatment prevalence. By contrast, among the highest prevalence (45–69% pre-treatment) villages, neither the C-C-C-C, C-C-S-S, S-S-S-S, nor WHO strategies yielded a long-term, post-treatment suppression of infection prevalence. Reinfection was rapid, and there was no obvious advantage among the four. In terms of relative costs, among the three most effective strategies for infection suppression (*i.e.*, C-C-C-C, C-C-S-S, or S-S-S-S), the S-S-S-S strategy was calculated to cost the least, based on differences in overall numbers treated (S-S-S-S = $4185, compared to $8455 and $6273 for C-C-C-C and C-C-S-S, respectively).

## Discussion

Results of our model's simulations indicate that in many moderate-to high prevalence areas, broad-based drug treatment programs will need to be sustained for four *or more* years to effect optimal suppression of local prevalence to <10%. Within the sub-district scale of our simulation, there were important differences in how the individual (albeit networked) communities responded to the treatment strategies that were implemented, and how long the impact of prevalence reduction could be maintained after suspension of treatment intervention. Programs with gaps in annual delivery (‘drug holidays’) were effective only in the lower prevalence villages, and did not fare as well as annual treatment programs (C-C-C-C, C-C-S-S, or S-S-S-S) in terms of long-term post-treatment suppression of infection prevalence.

For neglected tropical diseases ‘deworming’ campaigns, there has been a recognized need for optimal community uptake, sustainability, and continued political will to achieve the objectives of the program [52]. The findings of our calibrated simulation support this thinking. In particular, our analysis suggests that schistosomiasis control programs should anticipate the need for multi-year and even multi-decade programs in some villages; initially, to achieve maximal suppression of local prevalence (particularly in high-risk, high initial prevalence areas), and later, to maintain very low prevalence and maximal reduction in *Schistosoma*-associated disease. Intensive intervention in the initial years may result in prolonged suppression of infection in communities having low risk for reinfection, during which treatment (but, importantly, not surveillance) could be suspended. Program resources could then be re-purposed, and turned toward identification and retreatment of the highest risk villages. For now, these high risk villages are readily identified by their rapid re-emergence of infection prevalence (i.e., in 1–4 years). These same communities could also become the focus of non-drug interventions aiming at reduction of local transmission potential—in effect, re-forming high-risk villages into villages that have much lower risk of transmission and reinfection. Such efforts could include habitat modification at transmission sites, provision of safe alternatives for water use and recreation, and behavior change interventions focused on limiting water contamination and high-risk exposures.

Based on our model simulation, we found that a policy of annual rescreening of villages (with possible treatment reassignment according to current WHO 2006 recommendations [16]) before each yearly treatment can appear less costly if treatment adherence is initially high (>70%), but the associated results in terms of infection suppression are much less good (Table 3). This phenomenon is apparently related to a premature transition to alternate year therapy in truly high risk communities as they lower their prevalence into the ‘moderate’ category after the first round of treatment. For such high-risk communities, repeated annual treatment (for at least 4 years) appears to be necessary to reach fully effective suppression of infection prevalence.

There are clearly limitations to our analysis. Although our projections are based on model simulations that use an advanced stratified model that appears well calibrated for a specific region of southeastern Kenya, its projections may not be fully generalizable to other endemic areas. However, we believe that the modeled mosaic of high- and moderate- prevalence villages within a sub-district area is typical of many territories found within *Schistosoma*-endemic areas, and that the results of our simulation will prove valid for many other locations where schistosomiasis control proves to be challenging. Wherever school age children have the most important role in disease transmission in a given area, it is likely that the predictions for other geographic locations would be qualitatively similar to ours. Among the modeling assumptions that might influence the accuracy of our predictions are the following: i) we assume that treatment uptake is uniform across the program area and consistent from year to year. In fact, regular refusal or inability to participate, or progressive increases in non-adherence after initial years of treatment, could allow a core of untreated individuals to perpetuate transmission in a given sub-location [50], [53], [54]; ii) it is possible that repeated annual treatments may provide periodic boost to anti-schistosomal immunity and relative resistance to reinfection [55]. This feature, which could gradually reduce individual risk of reinfection, was not included in our study; iii) likewise, pond snail abundance was assumed to be stable each year over the period of the simulations. In drier landscapes where *Schistosoma* transmission is a rare event that is associated only with episodic flooding events, program response to therapy in high prevalence villages could be expected to be dramatically better than in our simulation, unless or until a flooding/transmission event again occurs. Of note, we have not modeled any effects of adjuvant snail control or other interventions aimed at reducing environmental transmission (this will be the focus of a forthcoming study). The focus of our present analysis was only the outcomes of different possible drug-based treatment interventions.

Given these caveats and limitations, we draw the following conclusions based on our modeling analysis:

- Community-wide or school-age targeted treatment programs will effectively limit transmission and reinfection in some but not all villages within a targeted treatment region.
- Overall level of treatment coverage will have an appreciable effect on the durability of infection suppression in a targeted multi-village area. Programs reaching >80% adherence will reach suppression faster, and those reaching >90% will have a much more prolonged impact in suppressing prevalence in component low-risk villages.
- That said, there will be a continuing need for regular, annual, program-based treatment in many high risk villages in order to maintain suppression of infection prevalence <10%.
- Although the entire area is treated in a given program, village-level factors, such as population density, lack of safe water and recreation options, and abundance of snail habitat, appear to favor perpetuation of reinfection risk much more than the network features of parasite transmission across the larger area.
- A longer duration of the initial treatment campaign (e.g., 4–8 years) will specifically benefit those areas where adherence is less good (60–70%). An early reassignment of communities to less intensive regimens after 1 or 2 years of initial success can reduce running costs, but appreciably impairs the ability of the program to effectively suppress infection prevalence, creating risk of more rapid reinfection and recurrent morbidity.
- At the village level, program response is strongly affected by pre-treatment village school age prevalence. Communities starting at ≤35% prevalence do well on shorter or intermittent treatment schedules. However, communities starting at >35% appear to require annual community or school age coverage for at least 4 years to attain good suppression of infection burden. SCORE trials for ‘gaining control’ among communities having >24% prevalence are expected to have mixed results when higher risk communities are given less than annual therapy for 4 years. We expect that in the parallel ‘sustaining control’ SCORE trials, which are being performed in communities starting at ≤24% prevalence, there should be nearly equivalent outcomes for all assigned schedules of treatment.
- The current WHO definition of ‘moderate’ prevalence (10–49%, Table 1) does not provide an effective decision point for treatment assignment, given much lower projections for treatment success in communities having prevalence >35%.

While these projected outcomes may not be fully realized in the seven ongoing SCORE operational trials, we feel that, for now, they offer useful, evidence-based, estimates of program outcomes where anti-schistosomal control programs are now being implemented. In terms of policy discussions and program design, the simulation results raise several new topics for consideration–There is clearly a need for ready identification of villages at high risk for reinfection. For now, annual rescreening of school age prevalence provides a basic marker of risk, but identification of other (proxy) features of high-risk villages could aid significantly in year-to-year planning for program deployment. In addition, beyond continuing surveillance, programs will need to decide how to manage their low-risk villages that no longer require therapy, and decide how best to bring them to the very desirable goal of complete transmission interruption.

## Supporting Information

Supplement S1 **Details of Stratified Worm Burden (SWB) modeling.**
(DOC)
