# Supplementary material for: Projecting the Long-Term Impact of School- or Community-Based Mass-Treatment Interventions for Control of Schistosoma Infection
Source: PLoS Negl Trop Dis. 2012 Nov 15;6(11):e1903. doi: 10.1371/journal.pntd.0001903 (PMC3499404; doi:10.1371/journal.pntd.0001903)
Supplement: Supplement S1 — Details of Stratified Worm Burden (SWB) modeling. (DOC) [file pntd.0001903.s001.doc]

**Supplemental Material: A Brief Description of the Stratified Worm Burden (SWB) Model**

The diagram of host strata and their potential transitions in *Stratified Worm Burden* (SWB) modeling approach for schistosomiasis, explained in detail in Gurarie, et al., 2010 is:

.

Here, the process of human infection from exposure to host snail-infested water, quantitatively represented by$\lambda$, brings people in the lower strata to higher strata, while worm death (at rate$\gamma_{i}$ ) does the opposite. Different strata *i* have specific resolution rates$\gamma_{i}$ that increase with burden, either due to density-dependent natural worm mortality or, possibly, experience related immunity. Additional source terms, *S*_i_ , represents new recruits to the infection pool (through newborns, migration into the area, and transitioning from younger to older age groups). Loss rates$\mu$ combine host mortality as well as out-migration, aging, etc. A differential equation system describing this scheme is:

The source term for children enters through an uninfected (zero) stratum of newborns each year, whereas the initial adult source is equal to older children’s SWB distribution, in that adults mostly come from local children through the process of aging. So, the source list for children is $\{S_{i}^{c}\}_{i=0}^{n}=\{bH_{a},0,\cdots,0\}$with $b$being birth rate and $H_{a}$adult population, and that for adult $\{S_{i}^{a}\}_{i=0}^{n}=\tau\frac{H_{c}}{H_{a}}\{h_{0}^{c},h_{i}^{c},\cdots,h_{n}^{c}\}$with $\tau$as transition rate from children to adults and $h_{i}^{c}$the $i$th stratum of child population.

In calibration of the village network model, the mean worm burden equations for a distributed system of *n* villages and *m* snail sites are as follows:

$$\begin{matrix} (\text{Human})\left\{\begin{matrix} \frac{dw_{i}^{c}}{dt}=A_{i}^{c}\text{ }\sum_{j=1}^{m} \eta_{ij}Z_{j}-(\gamma+\mu_{c}+\tau)w_{i}^{c}; \\ \frac{dw_{i}^{a}}{dt}=A_{i}^{a}\text{ }\sum_{j=1}^{m} \eta_{ij}Z_{j}+\tau\frac{H_{i}^{c}}{H_{i}^{a}}w_{i}^{c}-(\gamma+\mu_{a})w_{i}^{a};\left(i=1,...,n\right) \end{matrix}\right. \\ (\text{Worm})\left\{\begin{matrix} E_{i}^{c}=\rho^{c}w_{i}^{c}; \\ E_{i}^{a}=\rho^{a}w_{i}^{a};\left(i=1,...,n\right) \end{matrix}\right. \\ (\text{Snail})\left\{\begin{matrix} \frac{dY_{j}}{dt}=\Lambda_{j}\left(N_{j}-Y_{j}\right)-\nu Y_{j};\Lambda_{j}=B_{j}\sum_{i} \eta_{ij}(A_{i}^{c}H_{i}^{c}E_{i}^{c}+A_{i}^{a}H_{i}^{a}E_{i}^{a}) \\ Z_{j}=\psi_{j}Y_{j};\left(j=1,...,m\right) \end{matrix}\right. \end{matrix}$$

where $w_{i}^{s}$(*s* is either *c* or *a*, indicating children or adults) denotes the mean worm burden for children in village *i* , $w_{i}^{a}$for that of adults. Mean egg count, denoted by$E$, is set be proportional to female worm burden with an egg production rate$\rho$, which is taken as biological factor and is thus not site-specific. $N_{j}$,$Y_{j}$, and$Z_{j}$represent the local density of total, infected, and infectious snails, respectively. The snail transition rate from infected to infectious status depends on local environment, so we define it locally, denoted by $\psi_{j}$, where $j$is the index for a given snail site. We assume that the infection transmission rate from snails to human beings is proportional to the relative contact rate, $\eta_{ij}$, of a village, which is defined as the ratio of the number of visits to a specific snail site to the total number of visits to all snail sites for that village, so $A_{i}^{s}$is a local factor for establishment of transmission that needs to be estimated for each site. By the same pattern, snails at snail site *j* will get infected by humans from different village sites *i* in proportion to their absolute contact X that village’s human infection level (egg release). Connecting both with contact rate,$\eta_{ij}$, both transmission coefficients for snail infection from human group *s* are found to be proportional to$A_{i}^{s}$ with a local factor $B_{j}$(for more detail, please refer to ). This assumption reduces the number of unknown parameters to a manageable set $\left\{A_{i}^{c};A_{i}^{a};B_{j}\right\}$, consistent with the number of MWB equations.

In the past, when using mean worm burden as the metric for human infection intensity, chemotherapy treatment (praziquantel) was modeled as an additional loss in the mean worm burden over a time period. This additional loss rate can be derived from the basic pharmacodynamics. If we define the efficacy of a drug($e_{C}$) to be the fraction of worms killed by the drug within its effective duration and denote $T_{C}$be the effective duration of the chemotherapy, then the additional loss due to chemotherapy takes the following form:

$$-\frac{1}{T_{C}}\ln(1-e_{C})$$

This loss rate is added to the equations (for mean worm burden) only for the period of $T_{C}$since the additional loss rate is not valid for other times. In reality, we cannot cover the whole population or target subpopulation with chemotherapy because of limitations of drug delivery. So the target group is divided into two subgroups: covered and uncovered. For instance, if we choose a group of children for a drug treatment of efficacy $e_{C}$and effective duration $T_{C}$ , we use $W_{C}$to represent the mean worm burden of the covered children and $W_{U}$ for the uncovered subgroup. The differential equations for $W_{C}$is same as the uncovered group except the additional loss rate of worms. The mean worm burden of whole children group is$f_{C}W_{C}+(1-f_{C})W_{U}$, where $f_{C}$is the fraction of children treated by drug.

However, for modeling treatment effects with the SWB model, the effect of drug treatment on the prevalence of *Schistosoma* infection was implemented in the model by instantaneously shifting humans at higher burden levels (stratum) to lower levels according to the established efficacy of praziquantel . As an example, within the model, a treatment session with an efficacy of 90% (*i.e.,* killing 90% of worms) would bring a person in the 10^th^ stratum (i.e., one who is carrying between 90 and 100 worm pairs before therapy) down to the 0^th^ strata (carrying 0 to 10 worms), as the remaining number of worm pairs will most likely be between 9 and 10 of worms after the treatment. This is a more realistic effect than modeled in the traditional approaches used in the past, in which a treatment term is added to the natural mortality of worms to indicate an extra loss. In fact, the drug-killing effect on worms occurs more quickly (in less than one month) than natural death (~ 4-5 years ), and this more flexible scheme allows predictions for various timing and coverage among different control strategies, such as treating only the most heavily-infected groups.

In our model’s simulations we assumed at-random treatment coverage, in the sense that a random subset of each population was treated each time . In this sense, the ‘coverage’ of a control program means the fraction of all people treated in each treatment session, regardless of each individual’s past treatment history .

1. Gurarie D, King CH, Wang X (2010) A new approach to modelling schistosomiasis transmission based on stratified worm burden. Parasitology 137: 1951-1965.

2. Li Z (2008) Schistosomiasis Transmission and Control in A Distributed Heterogeneous Human-Snail Environment in Coastal Kenya. Cleveland: Case Western Reserve University. 69-70 p.

3. King CH, Mahmoud AAF (1989) Drugs 5 Years Later - Praziquantel. Annals of Internal Medicine 110: 290-296.

4. King CH, Muchiri EM, Ouma JH, 2000. Evidence against rapid emergence of praziquantel resistance in *Schistosoma haematobium*, Kenya. Emerging Infectious Diseases 6: 585-94.

5. Anderson RM, May RM (1991) Infectious Diseases of Humans. Dynamics and Control. New York: Oxford University Press. 467-496, 507-420 p.

6. Chan MS, Bundy DA (1997) Modelling the dynamic effects of community chemotherapy on patterns of morbidity due to *Schistosoma mansoni*. Trans R Soc Trop Med Hyg 91: 216-220.

7. Chan MS, Guyatt HL, Bundy DAP, Booth M, Fulford AJ, et al. (1995) The development of an age structured model for schistosomiasis transmission dynamics and control and its validation for *Schistosoma mansoni*. Epidemiol Infect 115: 325-344.

8. Black CL, Steinauer ML, Mwinzi PN, Evan Secor W, Karanja DM, et al. (2009) Impact of intense, longitudinal retreatment with praziquantel on cure rates of schistosomiasis mansoni in a cohort of occupationally exposed adults in western Kenya. Trop Med Int Health 14: 450-457.
